# Metabolic Dysregulation in ADHD: Implications for Appetite, Sleep, Stress Reactivity, and Pharmacological Treatment

**DOI:** 10.3390/metabo16080527

**Published:** 2026-07-27

**Authors:** Davoud Amiri, Swetang J. Shah, Lamberto Briziarelli, Sara Amiri, Barry Karlsson

**Affiliations:** 1 Private Neuropsychiatric Practice, 753 14 Uppsala, Sweden; 2 Bay Area Community Health (BACH), San Jose, CA 95127, USA; swetang.dr@gmail.com; 3 Department of Public Health, Faculty of Medicine, University of Perugia, 06123 Perugia, Italy; lamberto.brizia@gmail.com; 4 Department of Business Studies, Uppsala University, 753 14 Uppsala, Sweden; saraamiri1997@gmail.com; 5 Department of Medical Sciences, Uppsala University, 753 14 Uppsala, Sweden; barry.karlsson@live.se

**Keywords:** ADHD, hypothalamic inflammation, metabolic dysregulation, neuroinflammation, circadian rhythm, sleep disturbances, stress reactivity, HPA axis

## Abstract

**Background:** Attention-deficit/hyperactivity disorder (ADHD) has traditionally been understood through executive and frontostriatal models, but increasing evidence suggests that metabolic, inflammatory, circadian, and neuroendocrine mechanisms may also contribute to clinical heterogeneity. The hypothalamus is a central regulatory hub for appetite, sleep–wake organization, stress responsivity, autonomic function, and endocrine signalling. **Objective:** This review evaluated evidence linking ADHD with hypothalamic inflammation and related metabolic, inflammatory, circadian, and neuroendocrine dysregulation, with particular emphasis on appetite regulation, sleep and circadian function, stress reactivity, and pharmacological treatment response and tolerability. **Methods:** A systematic literature search was conducted in PubMed/MEDLINE, Embase, PsycINFO, Scopus, and Web of Science Core Collection from database inception to 31 May 2026. Human observational and intervention studies formed the primary evidence base. Evidence was synthesized narratively. Where available, quantitative findings from previously published meta-analyses were summarized to provide an overview of the strength and consistency of the evidence. **Results:** The reviewed evidence indicates that ADHD is associated with increased obesity risk, altered appetite-related hormones, immune-inflammatory changes, delayed circadian timing, atypical cortisol responsivity, and treatment-related effects on appetite, sleep, cardiovascular physiology, and tolerability. The strongest quantitative evidence concerned obesity, inflammatory markers, appetite hormones, and type 2 diabetes risk. Direct evidence for hypothalamic inflammation in ADHD remains limited, but converging indirect findings support hypothalamic and neuroendocrine mechanisms as biologically plausible contributors in a subgroup of individuals with ADHD. **Conclusions:** ADHD appears to be associated with broader metabolic, inflammatory, circadian, and neuroendocrine vulnerabilities beyond its core attentional and behavioural symptoms. Hypothalamic pathways should be interpreted as a mechanistic hypothesis rather than an established causal mechanism. Future longitudinal and multimodal studies are needed to clarify causality and identify biologically defined ADHD subgroups.

## 1. Introduction

Attention-deficit/hyperactivity disorder (ADHD) is a common neurodevelopmental disorder characterized by persistent symptoms of inattention, hyperactivity, and impulsivity that interfere with functioning across the lifespan. Although ADHD has traditionally been conceptualized in relation to executive dysfunction and frontostriatal dysregulation, increasing evidence indicates that broader biological systems may also contribute to its clinical expression and long-term health profile. In particular, metabolic, inflammatory, neuroendocrine, and circadian mechanisms have received growing attention because of their potential relevance to symptom variability, emotional regulation, physical health, and treatment tolerability [1,2,3,4,5].

Among the systems potentially relevant to ADHD, the hypothalamus occupies a unique position because it integrates metabolic, neuroendocrine, autonomic, and circadian signals. Consistent with this framework, a systematic review and meta-analysis has demonstrated altered task-related heart rate variability in individuals with ADHD, supporting disturbances in autonomic regulation [6]. As a central regulator of energy balance, appetite, sleep–wake cycles, stress responses, and endocrine function, the hypothalamus links peripheral physiological processes with neural circuits involved in cognition, motivation, and behaviour. Disturbances within these regulatory networks have therefore been proposed as a biologically plausible framework for understanding several clinical features frequently observed in ADHD, including altered eating behaviour, obesity, sleep disturbances, emotional dysregulation, and atypical stress responsivity [2,3,7].

Several lines of evidence support the relevance of metabolic dysregulation in ADHD. Epidemiological studies and meta-analyses have reported an association between ADHD and overweight or obesity across both pediatric and adult populations [1,8,9]. In the largest meta-analysis to date, ADHD was associated with significantly increased odds of obesity, with the association being particularly evident in adults [1]. These findings have encouraged interest in mechanisms that extend beyond lifestyle factors alone and include reward processing, appetite regulation, energy homeostasis, and endocrine signalling [1,7,10].

Recent studies have further suggested that hormones involved in appetite and energy regulation, including leptin, ghrelin, adiponectin, and related metabolic mediators, may contribute to ADHD symptomatology and executive dysfunction [7,10]. Such observations are biologically plausible given the extensive connections between hypothalamic nuclei, mesolimbic reward pathways, and prefrontal networks implicated in ADHD. Together, these findings raise the possibility that metabolic abnormalities may represent more than secondary consequences of behavioural symptoms and may instead reflect underlying neurobiological processes relevant to the disorder itself.

Interest has also increased in the role of inflammation and immune dysregulation in ADHD. Systematic reviews and meta-analyses indicate that individuals with ADHD may show altered peripheral inflammatory profiles, including changes in interleukin-6, tumour necrosis factor-alpha, and other immune-related markers, although findings remain heterogeneous [4,11,12]. More recent proteomic work has further suggested that an inflammatory biotype of ADHD may be linked to chronic stress exposure, supporting the possibility that immune and neuroendocrine mechanisms interact in at least a subgroup of affected individuals [5]. These observations do not establish inflammation as a primary cause of ADHD, but they provide a biologically plausible pathway through which chronic stress, immune activation, and metabolic regulation may converge.

The hypothalamic–pituitary–adrenal (HPA) axis represents another potentially important pathway. Stress regulation is closely linked to hypothalamic function, and alterations in cortisol secretion patterns have been reported in both children and adults with ADHD [11,12]. Meta-analytic evidence suggests that atypical stress responsivity may be present in at least a subgroup of affected individuals, although the direction and magnitude of findings vary across studies [11]. More recently, an inflammatory ADHD biotype associated with chronic stress exposure has been proposed, further supporting a possible interaction between inflammatory processes and neuroendocrine regulation [5].

Sleep and circadian disturbances are likewise highly prevalent in ADHD and may reflect underlying hypothalamic dysfunction. Systematic reviews have demonstrated consistent evidence of delayed sleep phase, evening chronotype preference, altered melatonin regulation, and disrupted circadian rhythms among individuals with ADHD [13,14,15]. Objective studies using dim-light melatonin onset, actigraphy, salivary biomarkers, and molecular circadian markers have identified delayed circadian timing and abnormalities in biological rhythm regulation that extend beyond subjective sleep complaints [14,15,16,17]. Because hypothalamic nuclei play a critical role in circadian synchronization, these findings provide an additional rationale for examining ADHD within a broader neurobiological framework that incorporates metabolic and neuroendocrine regulation.

Pharmacological treatment introduces further complexity. Stimulant medications remain the most effective treatment for core ADHD symptoms, yet they frequently influence appetite, weight, sleep, autonomic regulation, and other physiological processes that are closely linked to hypothalamic function [18,19,20]. Understanding the biological pathways underlying these effects may improve treatment individualization and contribute to a more comprehensive understanding of variability in treatment response and tolerability.

Despite increasing interest in these interconnected mechanisms, evidence remains dispersed across multiple disciplines, including psychiatry, endocrinology, sleep medicine, immunology, and metabolic research. To date, no comprehensive synthesis has specifically examined the extent to which hypothalamic inflammation and related metabolic dysregulation may contribute to appetite regulation, sleep and circadian function, stress reactivity, and pharmacological treatment outcomes in ADHD.

The aim of the present review was therefore to evaluate the evidence linking ADHD with hypothalamic inflammation and associated neuroendocrine, inflammatory, and metabolic dysregulation. Particular emphasis was placed on four clinically relevant domains: appetite and metabolic regulation, sleep and circadian function, stress reactivity, and pharmacological treatment response and tolerability. Where available, quantitative findings from previously published meta-analyses were summarized to provide an overview of the strength and consistency of the evidence.

## 2. Methods

### 2.1. Study Design and Reporting Framework

This review was based on a structured literature search. Where available, quantitative findings from previously published meta-analyses were summarized to provide an overview of the strength and consistency of the evidence. The review was based on a structured literature search designed to identify relevant human studies addressing the metabolic, inflammatory, circadian, neuroendocrine, and treatment-related aspects of ADHD. Human observational and intervention studies formed the primary evidence base. Preclinical findings were considered separately to clarify biological mechanisms but were not combined quantitatively with human data.

### 2.2. Eligibility Criteria and Framework

The eligibility criteria were developed using a PECO framework for observational studies and a complementary PICO framework for pharmacological intervention studies. This combined approach was selected because the review addressed both biological associations and treatment-related outcomes.

#### 2.2.1. Population

Eligible studies included children, adolescents, or adults with attention-deficit/hyperactivity disorder diagnosed according to recognised DSM or ICD criteria or through a clearly described structured clinical assessment. Studies based solely on self-reported ADHD symptoms were not included in the primary synthesis unless a validated instrument and an explicit diagnostic threshold had been used.

#### 2.2.2. Exposures and Biological Domains

For observational studies, eligible exposures included direct or indirect measures of hypothalamic, neuroendocrine, inflammatory, or metabolic dysregulation. Eligible exposures included:

- structural, functional, connectivity, or molecular measures involving the hypothalamus;
- circulating or central inflammatory markers, including cytokines, chemokines, acute-phase proteins, and related immune measures;
- hypothalamic–pituitary–adrenal axis activity and other neuroendocrine measures relevant to stress regulation;
- metabolic markers such as glucose, insulin, insulin resistance, lipid profiles, leptin, ghrelin, adiponectin, and other appetite- or energy-regulating hormones;
- metabolic syndrome, obesity, body mass index, or body-composition measures when examined in relation to ADHD and a relevant biological or pharmacological mechanism;
- sleep and circadian measures when investigated in relation to hypothalamic, inflammatory, neuroendocrine, or metabolic regulation.

Studies examining appetite, sleep, obesity, or stress in isolation were not considered sufficient unless the findings are linked to at least one of the biological domains specified above.

#### 2.2.3. Pharmacological Interventions

For intervention studies, eligible treatments included recognised stimulant and non-stimulant ADHD medications, including methylphenidate, amphetamine derivatives, atomoxetine, guanfacine, clonidine, and other pharmacological treatments used specifically for ADHD.

Studies may evaluate treatment initiation, discontinuation, dose change, duration of exposure, or comparisons between medication classes.

#### 2.2.4. Comparators

When applicable, comparators included:

- individuals without ADHD;
- healthy control participants;
- participants with ADHD without the metabolic, inflammatory, neuroendocrine, or hypothalamic alteration of interest;
- untreated or medication-naive participants with ADHD;
- pretreatment or within-subject baseline measurements;
- placebo or no-treatment conditions;
- alternative medication classes, doses, or durations of treatment;
- clinically relevant subgroups defined by age, sex, ADHD presentation, body mass index, obesity, sleep disturbance, or psychiatric comorbidity.

Studies without a separate comparator group may still be eligible when they use a longitudinal or within-subject design and report interpretable changes in relevant outcomes.

#### 2.2.5. Outcomes

The reviewed evidence was organized into four clinical domains:

1. Appetite and metabolic regulation, including appetite change, eating behaviour, food intake, body weight, body mass index, obesity, glucose regulation, insulin resistance, lipid metabolism, and metabolic syndrome.
2. Sleep and circadian function, including sleep duration, sleep quality, insomnia, daytime sleepiness, circadian rhythm, sleep–wake timing, and related physiological measures.
3. Stress reactivity and neuroendocrine regulation, including cortisol levels, diurnal cortisol patterns, stress-induced hormonal responses, hypothalamic–pituitary–adrenal axis function, and related physiological responses to stress.
4. Pharmacological treatment response and tolerability, including ADHD symptom response, appetite suppression, weight change, sleep disturbance, cardiovascular or metabolic adverse effects, treatment discontinuation, and changes in inflammatory, neuroendocrine, or metabolic markers during treatment.

Additional outcomes included emotional dysregulation, executive dysfunction, functional impairment, quality of life, and psychiatric or metabolic comorbidity when these outcomes were reported alongside an eligible biological or treatment-related measure.

### 2.3. Information Sources and Search Strategy

A literature search was conducted in PubMed/MEDLINE, Embase, PsycINFO, Scopus, and Web of Science Core Collection from database inception through 31 May 2026. Reference lists of eligible studies and relevant reviews were also screened to identify additional studies.

The search strategy combined controlled vocabulary (e.g., MeSH and Emtree, where applicable) with free-text terms related to ADHD, hypothalamic and neuroendocrine function, inflammation, metabolism, appetite regulation, sleep and circadian function, stress reactivity, and pharmacological treatment. The complete search strategies for all databases are provided in Supplementary Table S1.

### 2.4. Study Selection

All records identified through the electronic database searches were imported into reference-management software, and duplicate records were removed before screening.

Study selection was conducted in two stages. First, titles and abstracts were screened independently by two reviewers against the predefined eligibility criteria. Records judged potentially relevant by either reviewer proceeded to full-text assessment.

Second, the full texts of potentially eligible articles were evaluated independently by the same two reviewers. Reasons for exclusion at the full-text stage were recorded systematically. Disagreements at either stage were resolved through discussion and, when necessary, consultation with a third reviewer.

Before formal screening began, the reviewers piloted the eligibility criteria on a sample of records to ensure consistent interpretation of the inclusion and exclusion criteria. Any necessary clarifications were documented before the full screening process continued.

Studies reported in multiple publications were linked and treated as a single study when they described the same or overlapping participant sample. The most complete report was used as the primary source, while companion publications contributed additional outcomes or follow-up data where relevant. Participants were not counted more than once in any quantitative synthesis.

The study selection process is summarised in the PRISMA flow diagram, including records identified, duplicates removed, records screened, full-text reports assessed, exclusions with reasons, and studies included in the narrative synthesis [21].

### 2.5. Data Extraction and Data Management

Data were extracted using a structured data-extraction form developed for this review, with the data-extraction and management procedures informed by the JBI Manual for Evidence Synthesis [22]. The extracted information included study characteristics, population and comparator details, biological domains assessed, outcome measures, principal findings, quantitative estimates where available, and information relevant to methodological interpretation. The extraction form was refined during the initial review process to ensure that variables relevant to the review question were captured consistently.

- author, publication year, country, and study setting;
- study design and recruitment method;
- sample size and participant characteristics, including age, sex, diagnostic criteria, ADHD presentation, symptom severity, medication status, body mass index, obesity status, sleep disturbance, and relevant psychiatric or medical comorbidity;
- characteristics of the comparator group;
- hypothalamic, inflammatory, neuroendocrine, or metabolic variables assessed;
- methods, timing, and biological source of biomarker measurement;
- appetite-, weight-, metabolic-, sleep-, circadian-, and stress-related outcomes;
- pharmacological treatment characteristics, including medication class, dose, duration, treatment status, adherence, and comparator;
- treatment-response and tolerability outcomes;
- effect estimates and measures of variability;
- variables included in adjusted analyses;
- follow-up duration;
- information required for risk-of-bias assessment.

For studies reporting repeated measurements, data were extracted at all clinically relevant time points. Where several follow-up periods were available, short-, medium-, and long-term outcomes were retained separately where feasible.

When multiple measures were reported for the same outcome domain, preference was given to validated measures that most directly represented the prespecified outcome. Decisions regarding outcome selection were defined before quantitative synthesis and applied consistently across studies.

Where numerical data were presented only in figures, values were extracted using validated digital extraction methods when necessary. Estimated values were identified as such and examined in sensitivity analyses where appropriate.

Study authors were contacted when essential information was missing, unclear, or not reported in a form suitable for analysis. If clarification could not be obtained, the available data were used where interpretation remained possible, and the limitation was documented.

Disagreements between reviewers were resolved through discussion and, when necessary, consultation with a third reviewer. Any corrections made after comparison of the two extraction forms were documented.

Extracted data were stored in a structured electronic database with predefined variable names, coding rules, and version control. Each study was assigned a unique identification code. Publications based on overlapping samples were linked to prevent duplicate inclusion of participants or outcomes.

Before analysis, the dataset was checked for internal consistency, implausible values, duplicate entries, and discrepancies between extracted values and the original reports. A final verified dataset was retained for statistical analysis and preparation of evidence tables.

### 2.6. Risk-of-Bias Assessment

The methodological quality and risk of bias of the included studies were evaluated using study-design-appropriate assessment tools, including RoB 2 for randomized trials [23] and ROBINS-I for non-randomized intervention studies [24]. Risk-of-bias considerations informed the interpretation of the evidence, particularly when assessing consistency, methodological limitations, and the strength of the reported findings.

### 2.7. Prespecified Outcome Domains and Effect Measures

The outcomes were organised into four prespecified domains reflecting the main clinical and biological aims of the review.

#### 2.7.1. Prespecified Outcomes for Appetite and Metabolic Regulation

Outcomes in this domain included appetite, food intake, eating behaviour, weight change, body mass index, waist circumference, body composition, obesity, metabolic syndrome, fasting glucose, insulin, insulin resistance, glycated haemoglobin, lipid profiles, leptin, ghrelin, adiponectin, and other markers of energy balance or metabolic regulation.

#### 2.7.2. Prespecified Outcomes for Sleep and Circadian Function

Eligible outcomes included sleep duration, sleep onset latency, sleep efficiency, insomnia, daytime sleepiness, sleep quality, chronotype, circadian timing, melatonin-related measures, actigraphy findings, polysomnographic variables, and other measures of sleep–wake regulation.

#### 2.7.3. Prespecified Outcomes for Stress Reactivity and Neuroendocrine Regulation

Outcomes included basal cortisol, diurnal cortisol patterns, cortisol awakening response, stress-induced cortisol reactivity, hypothalamic–pituitary–adrenal axis measures, autonomic responses to stress, and other neuroendocrine markers directly relevant to stress regulation.

#### 2.7.4. Prespecified Outcomes for Treatment Response and Tolerability

Treatment-response outcomes included changes in ADHD symptom severity, global clinical improvement, executive functioning, emotional regulation, and functional outcomes.

Treatment-tolerability outcomes included appetite suppression, weight change, insomnia, daytime sleepiness, cardiovascular adverse effects, metabolic changes, neuroendocrine changes, inflammatory-marker changes, treatment discontinuation, dose reduction, and withdrawal due to adverse events.

Where studies reported several time points, outcomes were grouped, where feasible, as short-term, medium-term, and long-term follow-up.

### 2.8. Data Synthesis and Statistical Analysis

A structured narrative synthesis was conducted. Studies were grouped according to biological domain, study design, population, exposure or intervention, comparator, and outcome. Quantitative findings from previously published meta-analyses were summarised where available, but no new pooled meta-analysis was performed.

Findings were interpreted with attention to consistency across studies, study design, sample characteristics, biological plausibility, and clinical relevance. Evidence from preclinical studies was discussed separately and was not combined with human evidence. Quantitative estimates from existing meta-analyses were used only to contextualise the strength and direction of the available evidence.

### 2.9. Certainty of Evidence

The certainty of the available evidence was interpreted using considerations informed by the GRADE framework [25]. Separate judgements were made for the main outcome domains, including appetite and metabolic regulation, sleep and circadian function, stress reactivity and neuroendocrine regulation, and pharmacological treatment response and tolerability.

The assessment considered risk of bias, inconsistency, indirectness, imprecision, and publication bias. he interpretation of heterogeneity and potential publication bias was informed by established methodological recommendations for evidence synthesis and meta-analysis [26,27]. For observational evidence, additional consideration was given to the magnitude of association, dose–response relationships, and the likelihood that residual confounding could explain the observed findings.

The certainty of evidence was rated as high, moderate, low, or very low. Judgements were presented with brief justifications and interpreted in relation to the underlying study designs and the directness of the evidence.

### 2.10. Protocol Registration

No prospective protocol registration was performed for this review; therefore, no PROSPERO registration number is available [28].

## 3. Results

### 3.1. ADHD and Metabolic Dysregulation

Evidence from epidemiological, hormonal, and somatic health studies suggests that metabolic dysregulation is a clinically relevant feature of ADHD [1,6,10,17,29,30,31]. Across the available literature, findings consistently indicate associations between ADHD and obesity, altered appetite-regulating hormones, and increased rates of metabolic and somatic comorbidity [1,6,10,17,29,30,31].

The strongest evidence derives from the meta-analysis by Cortese et al. [1], which included 42 studies and 728,136 individuals. ADHD was significantly associated with obesity in both children (OR 1.20, 95% CI 1.05–1.37) and adults (OR 1.55, 95% CI 1.32–1.81). The prevalence of obesity was substantially higher among individuals with ADHD, particularly in adulthood, supporting a robust association between ADHD and adverse metabolic outcomes [1].

Beyond body weight alone, emerging evidence suggests that metabolic alterations may involve dysregulation of appetite-related endocrine pathways. Hsu et al. [6] reported significant associations between appetite hormone dysregulation and both ADHD symptom severity and executive dysfunction in adolescents with ADHD. These findings suggest that abnormalities in hormonal systems regulating satiety and energy balance may contribute to the cognitive and behavioural manifestations of the disorder.

Supporting this hypothesis, Özcan et al. [17] demonstrated altered circulating concentrations of leptin, adiponectin, and neuropeptide Y in drug-naïve children with ADHD. Because these hormones participate in hypothalamic regulation of appetite, energy expenditure, and reward-related feeding behaviour, their dysregulation provides a biologically plausible link between ADHD and metabolic disturbances.

A broader somatic perspective is provided by Instanes et al. [29], whose systematic review found that adults with ADHD exhibit increased rates of several medical disorders, including obesity-related conditions and metabolic disease. Additional evidence from studies on metabolic syndrome, insulin resistance, and type 2 diabetes further supports the relevance of metabolic vulnerability in ADHD [10,30,31,32].

Taken together, the available evidence indicates that ADHD is associated not only with behavioural risk factors for obesity but also with measurable alterations in biological systems involved in appetite regulation, energy homeostasis, and metabolic health. These findings support the hypothesis that hypothalamic and neuroendocrine mechanisms may contribute to the metabolic phenotype observed in a subgroup of individuals with ADHD [1,6,10,17,29,30,31].

### 3.2. Characteristics of Included Studies

The included studies covered five major biological domains: metabolic dysregulation, immune-inflammatory activation, sleep and circadian function, stress responsivity/HPA-axis function, and pharmacological treatment response or tolerability. Study designs included systematic reviews, meta-analyses, cohort studies, case–control studies, cross-sectional studies, and mechanistic investigations. The main characteristics of the included studies are summarized in Table 1.

### 3.3. Inflammation and Immune Activation

Evidence linking ADHD with immune-inflammatory dysregulation has expanded considerably over the past decade. Findings from systematic reviews, meta-analyses, molecular studies, and proteomic investigations suggest that at least a subgroup of individuals with ADHD may exhibit measurable alterations in inflammatory pathways [4,5,19,20].

Anand et al. [4] concluded that accumulating evidence supports a role for inflammatory processes in ADHD pathophysiology, although the literature remains heterogeneous. Proposed mechanisms include neuroimmune interactions during development, altered cytokine signalling, and shared inflammatory pathways across neuropsychiatric disorders.

More direct evidence comes from the meta-analysis by Misiak et al. [19], which demonstrated significantly elevated peripheral interleukin-6 concentrations in individuals with ADHD. The same analysis also identified reduced TNF-α levels, suggesting that inflammatory alterations in ADHD may not simply reflect generalized immune activation but rather a more complex pattern of immune dysregulation.

Similarly, Chang et al. [20] reported alterations in both cortisol-related and inflammatory biomarkers among youths with ADHD, further supporting interactions between immune and neuroendocrine systems.

The most recent evidence is provided by Schnorr et al. [5], who identified an inflammatory biotype of ADHD associated with chronic stress exposure. Their data-driven proteomic analysis suggests that inflammatory activation may characterize a biologically distinct subgroup of patients rather than the ADHD population as a whole.

Overall, the available evidence supports the presence of low-grade immune-inflammatory alterations in at least a subset of individuals with ADHD and provides a potential mechanistic link between stress exposure, neuroendocrine dysregulation, and metabolic dysfunction [4,5,19,20].

### 3.4. Sleep and Circadian Function

Sleep disturbances emerged as one of the most consistently reported biological correlates of ADHD. Across systematic reviews and mechanistic studies, evidence converged on alterations in circadian timing, chronotype preference, melatonin regulation, and sleep–wake organization. Collectively, these findings suggest that sleep-related abnormalities in ADHD extend beyond subjective complaints and may reflect disturbances in biological systems involved in circadian regulation [2,11,12,13,14,15,16].

The systematic review by Coogan and McGowan [2] identified consistent evidence of delayed circadian phase, evening chronotype preference, and altered circadian rhythm regulation across both pediatric and adult ADHD populations. The authors further noted that chronotherapeutic interventions targeting circadian timing may represent a potentially relevant treatment approach in selected patients.

Several objective investigations provided support for these observations. Baird et al. [11] reported alterations at behavioural, endocrine, and molecular levels, including abnormalities in salivary cortisol and melatonin rhythms, as well as the disrupted expression of circadian clock genes. These findings suggest that circadian dysregulation in ADHD may involve both central and peripheral biological timing systems.

Similarly, Bijlenga et al. [12] demonstrated delayed dim-light melatonin onset, delayed sleep onset, altered body-temperature rhythms, and shorter sleep duration among adults with ADHD and delayed sleep patterns. Objective actigraphic measures supported the presence of a biologically delayed circadian phase rather than simple behavioural sleep restriction.

Additional evidence was provided by Van Veen et al. [14], who found delayed circadian rhythms among adults with ADHD and chronic sleep-onset insomnia, reinforcing the concept that circadian phase delay may represent a common feature in a substantial subgroup of patients. Earlier work by Van der Heijden et al. [15] similarly suggested that chronic sleep-onset insomnia in ADHD frequently displays characteristics of a circadian rhythm sleep disorder rather than primary insomnia alone. In a subsequent trial, melatonin treatment improved sleep timing in children with ADHD and chronic sleep-onset insomnia, supporting the clinical relevance of circadian mechanisms in this subgroup [16].

Finally, Bumb et al. [13] reported associations between ADHD symptom severity, evening chronotype preference, and reduced pineal gland volume. Although exploratory in nature, these findings raise the possibility that structural differences within biological systems involved in melatonin regulation may contribute to circadian abnormalities observed in ADHD.

Taken together, the available evidence supports the presence of clinically meaningful circadian disturbances in ADHD, characterized primarily by delayed biological timing, eveningness, and altered melatonin-related regulation. These findings are consistent with the broader hypothesis that neuroendocrine and hypothalamic mechanisms contribute to sleep dysfunction in ADHD [2,11,12,13,14,15,16].

### 3.5. Stress Reactivity and HPA Axis Function

Evidence regarding stress responsivity in ADHD suggests abnormalities in neuroendocrine regulation, although findings have been more heterogeneous than those observed for circadian disturbances [3,11,20].

The most comprehensive synthesis was provided by Kamradt et al. [3], who reported significant alterations in cortisol reactivity following experimental stressors in individuals with ADHD. Across studies, ADHD was associated with atypical physiological responses to stress, supporting the notion that HPA-axis regulation differs from that observed in unaffected individuals. However, substantial between-study variability indicated that these abnormalities may not be present across all ADHD populations.

Further evidence was reported by Chang et al. [20], whose systematic review and meta-analysis identified alterations in both cortisol-related and inflammatory biomarkers among youths with ADHD. These findings suggest that neuroendocrine and immune pathways may be interconnected rather than operating independently.

At a more mechanistic level, Baird et al. [11] observed altered salivary cortisol rhythms alongside circadian abnormalities in adults with ADHD. The coexistence of circadian and stress-related alterations suggests that disturbances in biological timing systems may contribute to dysregulated HPA-axis functioning.

More recently, Schnorr et al. [5] identified a distinct inflammatory biotype of ADHD characterized by proteomic signatures associated with chronic stress exposure. Their findings suggest that chronic stress may interact with immune activation in a subgroup of individuals with ADHD, providing a potential biological framework linking inflammation, neuroendocrine dysregulation, and clinical heterogeneity.

Overall, the available evidence supports altered stress responsivity and HPA-axis functioning in at least a subset of individuals with ADHD. Although the direction and magnitude of cortisol abnormalities vary across studies, converging evidence suggests that interactions between chronic stress, inflammation, and neuroendocrine regulation may contribute to the broader biological phenotype of ADHD [3,5,11,20].

### 3.6. Pharmacological Treatment Response and Tolerability

Available pharmacological studies demonstrated that ADHD medications influence several physiological domains relevant to appetite regulation, metabolic function, autonomic activity, and long-term health outcomes.

The largest comparative evaluation was conducted by Cortese et al. [8], whose network meta-analysis confirmed that stimulant medications remain among the most effective treatments for core ADHD symptoms across age groups. At the same time, treatment efficacy was accompanied by predictable tolerability profiles, including appetite suppression and sleep-related adverse effects, particularly with stimulant compounds.

Evidence regarding appetite-related adverse effects was further strengthened by the meta-analysis of Holmskov et al. [9] During methylphenidate treatment, children and adolescents showed significantly increased risks of reduced appetite, weight loss, and gastrointestinal adverse events, including abdominal discomfort. These findings indicate that treatment-related effects on feeding behaviour and energy balance are common and clinically relevant.

Although most treatment studies have focused on symptom reduction rather than metabolic outcomes, these adverse-effect profiles are notable because they involve physiological systems regulated, at least in part, by hypothalamic pathways controlling appetite, satiety, and energy homeostasis.

Long-term safety data were provided by Zhang et al. [18], who examined cardiovascular outcomes associated with ADHD medication exposure. Their findings suggested a modest increase in cardiovascular disease risk with prolonged cumulative treatment exposure, highlighting the importance of ongoing monitoring in patients receiving long-term pharmacotherapy.

Taken together, the available evidence suggests that ADHD medications influence biological systems extending beyond symptom control, including pathways involved in appetite regulation, metabolism, sleep, and autonomic function. These findings highlight the importance of considering neuroendocrine and metabolic factors when evaluating treatment response and tolerability in ADHD.

Key quantitative findings across biological domains are summarized in Table 2.

**Figure 1:**
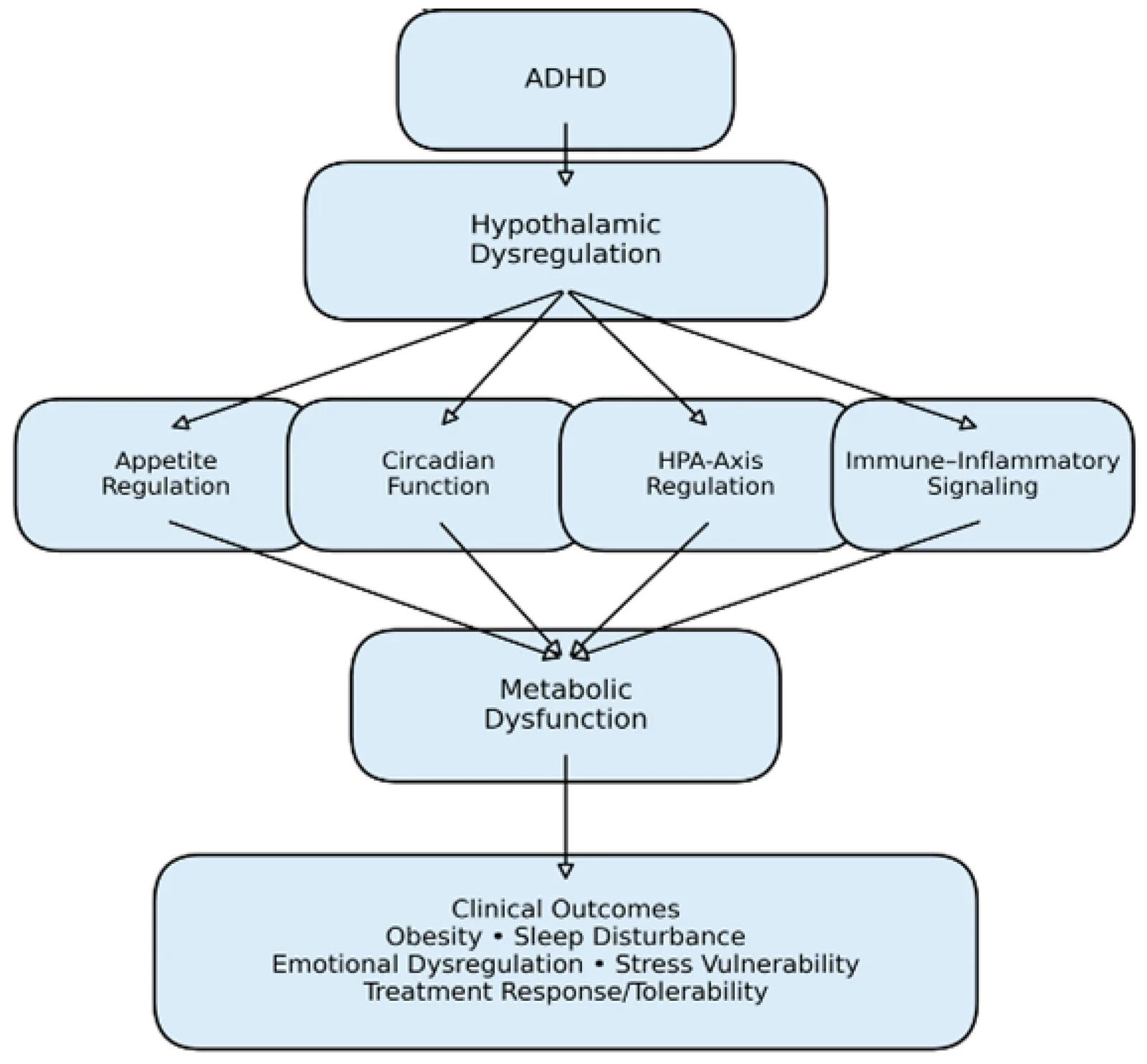
Conceptual model of hypothalamic and metabolic dysregulation in ADHD.

The conceptual framework integrating the principal biological pathways discussed in this review is illustrated in Figure 1.

## 4. Discussion

This systematic review suggests that ADHD is associated with alterations across several interconnected biological domains, including metabolic regulation, appetite-related hormones, immune-inflammatory activity, circadian function, stress responsivity, and treatment tolerability. The findings do not support a single explanatory pathway for ADHD. Rather, they suggest that a subgroup of individuals with ADHD may exhibit broader neuroendocrine and metabolic vulnerability that extends beyond core attentional and behavioural symptoms [1,2,3,4,5,6,7,8,9,10,11,12,13,14,15,16,17,18,19,20,29,30,31].

A central finding of this review is that metabolic dysregulation appears to be clinically relevant in at least a subgroup of individuals with ADHD. Evidence from epidemiological studies, systematic reviews, and meta-analyses supports associations between ADHD and obesity, altered appetite regulation, metabolic syndrome, insulin resistance, and diabetes-related outcomes [1,6,10,17,29,30,31,33]. Although impulsive eating, impaired planning, sleep disturbance, and reduced physical activity may contribute to these associations, the available evidence also suggests that biological mechanisms involving hypothalamic regulation, neuroendocrine signalling, and metabolic homeostasis are likely to play an important role.

**Figure 2:**
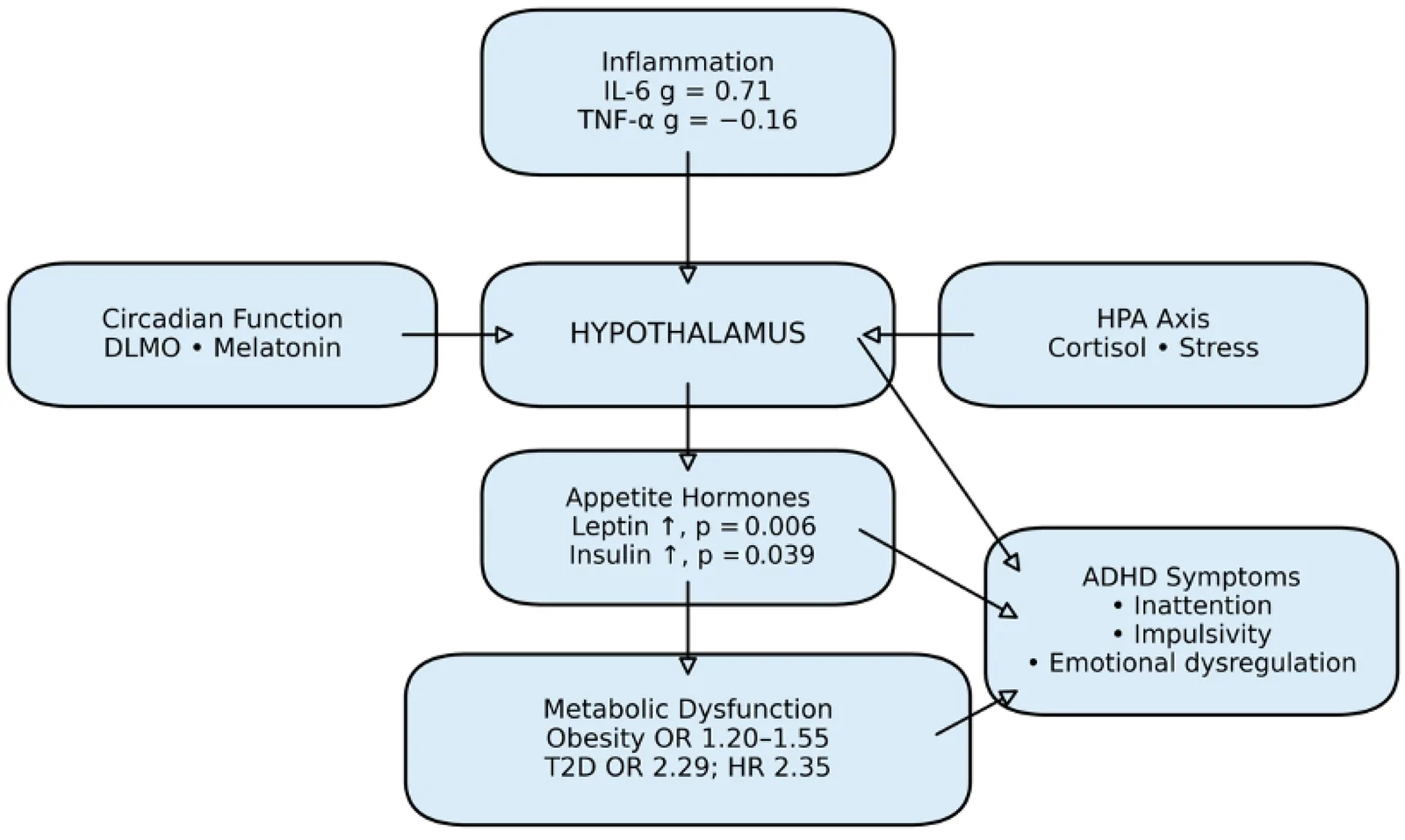
Integrated pathophysiological framework linking inflammation, neuroendocrine regulation, circadian function, and metabolic dysfunction in ADHD.

Figure 2 summarizes the proposed integrative framework linking inflammatory activation, circadian dysregulation, HPA-axis alterations, appetite-related hormones, and metabolic dysfunction in ADHD.

Inflammation provides a second biologically plausible link. Systematic reviews and meta-analyses indicate that inflammatory markers may be altered in at least a subset of individuals with ADHD, although findings remain heterogeneous [4,19,20]. The more recent identification of an inflammatory biotype associated with chronic stress exposure further supports the possibility that immune activation may characterize a subgroup rather than the entire ADHD population [5]. This distinction is clinically important because it avoids treating inflammation as a universal explanation for ADHD while still allowing it to be considered as a relevant mechanism in selected individuals.

The hypothalamus may provide a useful integrative framework for these findings. It is centrally involved in appetite regulation, sleep–wake organization, stress responses, autonomic function, and neuroendocrine signalling. Hypothalamic inflammation and microglial activation have been implicated in metabolic disease and obesity, and these mechanisms may help explain. Hypothalamic inflammation and microglial activation have been implicated in metabolic disease and obesity, and these mechanisms may help explain how chronic stress, metabolic vulnerability, and immune activation can converge biologically [34,35,36,37,38,39,40]. In the present context, hypothalamic involvement should be interpreted as a mechanistic hypothesis rather than an established causal pathway in ADHD.

Sleep and circadian findings were among the most consistent observations. Delayed circadian phase, evening chronotype preference, altered melatonin regulation, and biological rhythm abnormalities have been reported across several studies [2,11,12,13,14,15,16]. These findings are clinically relevant because circadian disruption may aggravate emotional regulation, appetite control, stress tolerance, and treatment response. They also support routine assessment of sleep timing and biological rhythm disturbances in patients with ADHD.

Treatment studies add further complexity. ADHD medications remain effective for core symptoms, but they also influence appetite, weight, sleep, cardiovascular physiology, and tolerability profiles [8,9,18]. These effects are often considered adverse events, but they may also reveal underlying biological systems involved in appetite regulation, autonomic balance, and neuroendocrine sensitivity. A broader biological assessment may therefore improve clinical understanding of why some patients respond well to treatment, whereas others develop appetite suppression, sleep disturbance, cardiovascular symptoms, or reduced tolerability.

Overall, the available evidence supports a cautious but coherent model in which metabolic, inflammatory, circadian, and stress-related abnormalities may interact in a subgroup of individuals with ADHD. This model does not replace established neurodevelopmental explanations of ADHD. Instead, it expands them by highlighting biological systems that may contribute to clinical heterogeneity, somatic comorbidity, and differences in treatment response.

## 5. Conclusions

ADHD is increasingly recognized as a neurodevelopmental disorder with relevant metabolic, neuroendocrine, circadian, and inflammatory correlates. The evidence reviewed here suggests that alterations in appetite regulation, stress responsivity, biological rhythms, immune function, and metabolic homeostasis may contribute to clinical heterogeneity and somatic comorbidity in at least a subset of individuals with ADHD.

Although current evidence does not establish a direct causal role for hypothalamic inflammation in ADHD, available findings support the hypothesis that hypothalamic pathways may represent a biological interface linking stress regulation, sleep, appetite, metabolism, and treatment response. This perspective may help explain why individuals with ADHD differ substantially in symptom presentation, physical health outcomes, and medication tolerability.

Future longitudinal and mechanistic studies are needed to clarify causal relationships and to determine whether biologically informed subgroups can be identified. A better understanding of these interconnected systems may ultimately contribute to more individualized assessment strategies and targeted therapeutic approaches in ADHD.

## 6. Limitations

This review has several limitations that should be acknowledged. First, the available literature remains heterogeneous with respect to study design, participant characteristics, age groups, outcome measures, and biological domains investigated. Second, many included studies were observational or cross-sectional, limiting conclusions regarding causality. Third, relatively few studies directly examined hypothalamic inflammation, and much of the current evidence is derived from indirect markers involving circadian regulation, stress responsivity, metabolic function, or peripheral inflammatory processes. Fourth, variability in medication exposure, psychiatric comorbidity, obesity status, and lifestyle factors may have contributed to between-study differences. Finally, publication bias and selective reporting cannot be fully excluded despite systematic study identification and quality assessment procedures.

## 7. Risk of Bias Considerations

The overall quality of the evidence varied across biological domains. Several studies were limited by modest sample sizes, cross-sectional designs, incomplete control of confounding variables, and differences in biomarker assessment methods. Furthermore, inflammatory, metabolic, and neuroendocrine markers are influenced by multiple environmental and clinical factors, which may affect comparability across studies. Consequently, the findings should be interpreted as evidence of biological associations rather than definitive proof of causal mechanisms linking hypothalamic dysfunction to ADHD.

## 8. Future Research Directions

Future research should prioritize prospective longitudinal studies capable of clarifying temporal and causal relationships among hypothalamic function, inflammation, metabolic dysregulation, circadian disturbances, and ADHD symptomatology. Greater methodological standardization of biological measurements and outcome definitions would improve comparability across studies. Further investigation of biologically defined ADHD subgroups may help identify distinct pathophysiological mechanisms and differential treatment responses. In addition, multimodal approaches integrating neuroimaging, endocrine markers, inflammatory biomarkers, metabolic assessments, and clinical phenotyping may provide a more comprehensive understanding of the biological heterogeneity of ADHD.

## Figures and Tables

**Table 1:** **Characteristics of Included Studies.** This table summarizes the main characteristics of the studies included in the review, including study design, population, biological domain, and principal finding.

Study	Year	Design	Population/Sample	Biological Domain	Principal Finding
Cortese et al. [1]	2016	Systematic review/meta-analysis	728,136 participants	Obesity/metabolism	ADHD associated with increased obesity risk in children and adults.
Kamradt et al. [3]	2018	Meta-analysis	ADHD + controls; mixed age groups	HPA-axis/stress reactivity	Altered cortisol reactivity following experimental stressors.
Misiak et al. [19]	2022	Systematic review/meta-analysis	10 studies; ADHD + controls	Inflammation	Increased IL-6 and altered TNF-alpha concentrations.
Chang et al. [20]	2021	Systematic review/meta-analysis	Youth with ADHD	HPA-axis/inflammation	Altered cortisol-related and inflammatory biomarkers.
Schnorr et al. [5]	2024	Proteomic study	ADHD cohort	Stress/inflammation	Inflammatory biotype associated with chronic stress exposure.
Hsu et al. [7]	2023	Cross-sectional study	Adolescents with ADHD	Appetite hormones	Insulin and leptin dysregulation associated with symptom and executive-function measures.
Ozcan et al. [17]	2018	Case–control study	Drug-naive ADHD children	Appetite hormones	Altered leptin, adiponectin, and neuropeptide Y profile.
Coogan & McGowan [2]	2017	Systematic review	ADHD populations across the lifespan	Circadian function	Eveningness, delayed chronotype, and circadian disruption described across ADHD populations.
Baird et al. [11]	2012	Case–control study	Adults with ADHD	Circadian biology	Altered melatonin, cortisol, and circadian clock-gene markers.
Bijlenga et al. [12]	2013	Case–control study	Adults with ADHD	Circadian function	Delayed dim-light melatonin onset and delayed sleep-phase pattern.
Van Veen et al. [14]	2010	Case–control study	Adults with ADHD	Circadian function	Delayed circadian rhythm and sleep-onset insomnia.
Van der Heijden et al. [15]	2005	Observational study	Pediatric ADHD	Sleep/circadian function	Circadian sleep-onset delay reported in children with ADHD.
Van der Heijden et al. [16]	2007	Intervention study	Pediatric ADHD	Sleep treatment	Melatonin improved sleep timing in children with ADHD and sleep-onset insomnia.
Landau & Pinhas-Hamiel [10]	2019	Narrative review	ADHD populations	Metabolic syndrome	ADHD discussed as a group with increased cardiometabolic vulnerability.
Garcia-Argibay et al. [30]	2023	Systematic review/meta-analysis + sibling study	ADHD/T2D cohorts; 5.7 million individuals	Type 2 diabetes	ADHD associated with increased type 2 diabetes risk.
di Girolamo et al. [31]	2022	Cross-sectional study	Adult ADHD outpatients	Metabolic syndrome	Increased metabolic burden and insulin-resistance-related findings.
Fanelli et al. [32]	2022	Genetic study	Large genetic cohorts	Insulin signalling	Shared metabolic-psychiatric genetic architecture involving insulin-related traits.
Holmskov et al. [9]	2017	Meta-analysis	Methylphenidate-treated ADHD patients	Treatment tolerability	Increased appetite suppression and gastrointestinal adverse events.
Zhang et al. [18]	2024	Population cohort	ADHD medication users	Treatment safety	Long-term cardiovascular risk signal with prolonged ADHD medication exposure.

Abbreviations: ADHD, attention-deficit/hyperactivity disorder; HPA, hypothalamic–pituitary–adrenal; IL-6, interleukin-6; T2D, type 2 diabetes; TNF-alpha, tumor necrosis factor-alpha.

**Table 2:** Quantitative Summary of Key Findings Across Biological Domains. This table summarizes the principal quantitative and clinically relevant findings reported across the biological domains included in the review.

Biological Domain	Biomarker/Outcome	Key Finding	Representative Study
Obesity/metabolism	Childhood obesity	OR 1.20 (95% CI 1.05–1.37)	Cortese et al., 2016 [1]
Obesity/metabolism	Adult obesity	OR 1.55 (95% CI 1.32–1.81)	Cortese et al., 2016 [1]
Obesity prevalence	Adults with ADHD vs. controls	28.2% vs. 16.4%	Cortese et al., 2016 [1]
Obesity prevalence	Children with ADHD vs. controls	10.3% vs. 7.4%	Cortese et al., 2016 [1]
Inflammation	IL-6	g = 0.71 (95% CI 0.12–1.31)	Misiak et al., 2022 [19]
Inflammation	TNF-alpha	g = −0.16 (95% CI −0.30 to −0.03)	Misiak et al., 2022 [19]
Appetite hormones	Insulin	Increased; *p* = 0.039	Hsu et al., 2023 [7]
Appetite hormones	Leptin	Increased; *p* = 0.006	Hsu et al., 2023 [7]
Executive function	Insulin/executive-function association	*p* = 0.031	Hsu et al., 2023 [7]
Type 2 diabetes	ADHD and T2D risk	OR 2.29 (95% CI 1.48–3.55)	Garcia-Argibay et al., 2023 [30]
Type 2 diabetes	Swedish cohort estimate	HR 2.35 (95% CI 2.14–2.58)	Garcia-Argibay et al., 2023 [30]
Circadian function	Delayed circadian rhythm/melatonin markers	Delayed DLMO, eveningness, and altered sleep phase reported	Coogan & McGowan [2], Baird [11], Bijlenga [12], Van Veen [14]
HPA-axis/stress	Cortisol reactivity	Atypical cortisol response patterns reported across stress paradigms	Kamradt et al., 2018 [3]; Chang et al., 2021 [20]
Treatment tolerability	Methylphenidate-related appetite/GI effects	Increased appetite suppression and gastrointestinal adverse events	Holmskov et al., 2017 [9]
Treatment safety	Long-term ADHD medication exposure	Increased cardiovascular risk signal with longer cumulative exposure	Zhang et al., 2024 [18]

Abbreviations: ADHD, attention-deficit/hyperactivity disorder; CI, confidence interval; DLMO, dim-light melatonin onset; GI, gastrointestinal; HR, hazard ratio; IL-6, interleukin-6; OR, odds ratio; T2D, type 2 diabetes; TNF-alpha, tumor necrosis factor-alpha.

## Data Availability

No new datasets were generated or analyzed during this study. All data supporting the findings of this review are available from the cited published literature.

## Supplementary Materials

The following supporting information can be downloaded at: https://www.mdpi.com/article/10.3390/metabo16080527/s1, Table S1: Complete Study Matrix of Included Studies.
