# Supplementary material for: Metabolic Dysregulation in ADHD: Implications for Appetite, Sleep, Stress Reactivity, and Pharmacological Treatment
Source: Metabolites. 2026 Jul 27;16(8):527. doi: 10.3390/metabo16080527 (PMC13515411; doi:10.3390/metabo16080527)
Supplement: Supplementary file 1 [file metabolites-16-00527-s001.zip › metabolites-4417296-supplementary.pdf]

Supplementary Table S1. Complete Study Matrix of Included Studies

This supplementary table provides the complete study matrix, including study design, population, age group, sample size, biological domain, biomarker or exposure, main outcome, key finding, and risk-of-bias assessment.

Study	Year	Design	Population	Age group	N/sample	Biological domain	Main biomarker/exposure	Main outcome	Key finding	Risk of bias
Cortese et al.	2016	Systematic review/meta-analysis	ADHD + controls	Children/adults	728,136	Obesity/metabolism	BMI/obesity	Obesity risk	ADHD associated with increased obesity risk	Moderate
Kamradt et al.	2018	Meta-analysis	ADHD + controls	Mixed	NR	HPA-axis	Cortisol reactivity	Stress response	Altered cortisol reactivity	Moderate
Misiak et al.	2022	Systematic review/meta-analysis	ADHD + controls	Mostly youth	10 studies	Inflammation	IL-6, TNF-alpha, CRP	Cytokine differences	Increased IL-6 and altered TNF-alpha	Moderate
Chang et al.	2021	Systematic review/meta-analysis	Youth with ADHD	Youth	NR	HPA-axis/inflammation	Cortisol, inflammatory markers	Biomarker alterations	Altered cortisol/inflammatory profile	Moderate
Schnorr et al.	2024	Proteomic study	ADHD cohort	Mixed	NR	Stress/inflammation	Inflammatory proteome	ADHD biotype	Inflammatory biotype linked to chronic stress	Moderate
Hsu et al.	2023	Cross-sectional	Adolescents with ADHD	Adolescents	NR	Appetite hormones	Insulin, leptin	Symptoms/executive function	Insulin/leptin dysregulation	Moderate
Özcan et al.	2018	Case-control	Drug-naive ADHD children	Children	NR	Appetite hormones	Leptin, adiponectin, NPY	Hormone levels	Altered appetite hormone profile	Moderate
Coogan & McGowan	2017	Systematic review	ADHD populations	Lifespan	NR	Circadian function	Chronotype/circadian markers	Sleep/circadian rhythm	Eveningness and circadian disruption	Moderate
Baird et al.	2012	Case-control	Adult ADHD	Adults	NR	Circadian biology	Melatonin, cortisol, PER2/BMAL1	Biological rhythms	Altered circadian rhythm markers	Moderate
Bijlenga et al.	2013	Case-control	Adult ADHD	Adults	NR	Circadian function	DLMO, actigraphy, temperature	Sleep phase	Delayed circadian phase	Moderate
Van Veen et al.	2010	Case-control	Adult ADHD	Adults	NR	Circadian function	Circadian rhythm markers	Sleep-onset insomnia	Delayed circadian rhythm	Moderate
Van der Heijden et al.	2005	Observational	Pediatric ADHD	Children	NR	Sleep/circadian	Sleep-onset timing	Insomnia	Circadian sleep-onset delay	Moderate
Van der Heijden et al.	2007	Intervention	Pediatric ADHD	Children	NR	Sleep treatment	Melatonin	Sleep/behaviour/cognition	Melatonin improved sleep timing	Moderate
Landau & Pinhas-Hamiel	2019	Review	ADHD populations	Mixed	NR	Metabolic syndrome	Obesity, T2D, hypertension	Cardiometabolic risk	ADHD as cardiometabolic risk group	Low-moderate
Garcia-Argibay et al.	2023	Systematic review/meta-analysis + sibling study	ADHD/T2D cohorts	Mixed	5.7 million	Type 2 diabetes	T2D diagnosis	Diabetes risk	Increased T2D risk	Moderate
di Girolamo et al.	2022	Cross-sectional	Adult ADHD outpatients	Adults	NR	Metabolic syndrome	Insulin resistance	Metabolic syndrome	Increased metabolic burden	Moderate
Fanelli et al.	2022	Genetic study	Large genetic cohorts	Mixed	NR	Insulin signalling	Genetic overlap	Insulin-related traits	Shared metabolic-psychiatric architecture	Moderate
Holmskov et al.	2017	Meta-analysis	MPH-treated ADHD	Children/adolescents	NR	Treatment tolerability	Methylphenidate	GI/appetite adverse events	Appetite and GI adverse effects	Moderate
Zhang et al.	2024	Population cohort	ADHD medication users	Mixed	NR	Treatment safety	ADHD medication exposure	Cardiovascular disease	Long-term cardiovascular risk signal	Moderate

Abbreviations: ADHD, attention-deficit/hyperactivity disorder; BMI, body mass index; CRP, C-reactive protein; DLMO, dim light melatonin onset; GI, gastrointestinal; HPA, hypothalamic-pituitary-adrenal; IL-6, interleukin-6; MPH, methylphenidate; NPY, neuropeptide Y; NR, not reported in the original study or extraction file; T2D, type 2 diabetes; TNF-alpha, tumor necrosis factor-alpha.
